# A deprescribing programme aimed to optimise blood glucose-lowering medication in older people with type 2 diabetes mellitus, the OMED2-study: the study protocol for a randomised controlled trial

**DOI:** 10.1186/s13063-024-08249-9

**Published:** 2024-07-25

**Authors:** Charlotte Andriessen, Marieke T. Blom, Beryl A. C. E. van Hoek, Anna W. de Boer, Petra Denig, G. Ardine de Wit, Karin Swart, Angela de Rooij-Peek, Rob J. van Marum, Jacqueline G. Hugtenburg, Pauline Slottje, Daniël van Raalte, Liselotte van Bloemendaal, Ron Herings, Giel Nijpels, Rimke C. Vos, Petra J. M. Elders

**Affiliations:** 1 Department of General Practice, Amsterdam UMC, Location Vrije Universiteit, Meibergdreef 15, Amsterdam, 1105AZ the Netherlands; 2 Amsterdam Public Health Research Institute, Amsterdam, the Netherlands; 3 Department of Public Health and Primary Care / Health Campus The Hague, LUMC, Leiden, The Netherlands; 4 Department of Clinical Pharmacy and Pharmacology, University of Groningen, University Medical Center Groningen, Groningen, Netherlands; 5 Department of Health Sciences, Faculty of Beta Sciences, Vrije Universiteit Amsterdam, Amsterdam, the Netherlands; 6 Centre for Public Health, Healthcare and Society, National Institute of Public Health and the Environment, Bilthoven, the Netherlands; 7 PHARMO Institute for Drug Outcomes Studies, Utrecht, The Netherlands; 8 Diabetesvereniging Nederland, Leusden, Netherlands; 9 Department of Geriatric Medicine, Jeroen Bosch Hospital, ‘s-Hertogenbosch, the Netherlands; 10 Department of Clinical Pharmacology, Jeroen Bosch Hospital, ‘s-Hertogenbosch, the Netherlands; 11 Department of Elderly Care Medicine, Amsterdam University Medical Center, Amsterdam, the Netherlands; 12 Department of General Practice, Julius Center for Health Sciences and Primary Care, University Medical Center Utrecht, Utrecht University, Utrecht, the Netherlands; 13 Diabetes Center, Department of Internal Medicine, Amsterdam University Medical Center, Amsterdam, Netherlands; 14 Department of Internal Medicine - Geriatrics, Amsterdam University Medical Center, Amsterdam, Netherlands

**Keywords:** Elderly, Type 2 diabetes, Overtreatment, Hypoglycaemia, General practitioners, Extended normalisation process theory

## Abstract

**Background:**

Older patients with type 2 diabetes mellitus (T2D) have an increased risk of hypoglycaemic episodes when using sulphonylureas or insulin. In the Netherlands, guidelines exist for reducing glucose-lowering medication in older patients. However, evidence is lacking that a medication reduction in older patients can be safely pursued. Here, we will examine if promoting the deprescribing of insulin/sulphonylureas with a deprescribing programme (DPP) in general practice affects T2D-complications in older overtreated patients.

**Methods:**

We will perform a 1:1 cluster randomised controlled trial in 86 general practices in the Netherlands. The DPP will consist of education sessions with general practitioners and practice nurses about reducing glucose-lowering medication in older patients (≥ 70 years). Topics of the sessions include the necessity of deprescribing, tools to initiate deprescribing and strategies to discuss deprescribing with patients (shared decision making). The DPP further includes a support programme with practice visits. The study will employ a selection tool to identify possibly overtreated older patients from the electronic medical records of the general practitioner. Eligibility for enrolment in the study will be based on HbA1c targets indicated by the Dutch guidelines, which depend on age, diabetes duration, presence of frailty, and life expectancy. The control group will provide usual care. We aim to include 406 patients. The follow-up period will be 2 years. For the primary outcome, the effect of the DPP on T2D-complications will be assessed by counting the cumulative incidence of events related to under- and overtreatment in T2D as registered in the electronic medical records. We shall perform an intention-to-treat analysis and an analysis including only patients for whom deprescribing was initiated. The implementation of the DPP in general practice will be evaluated quantitatively and qualitatively using the Extended Normalisation Process Theory (ENPT) and the Reach, Efficacy – Adoption, Implementation and Maintenance (RE-AIM) model. Other secondary outcomes include quality of life, cognitive functioning, events related to overtreatment or undertreatment, biomarkers of health, amount of blood glucose-lowering medication prescriptions, and cost-effectiveness.

**Discussion:**

This study will provide insight into the safety and feasibility of a programme aimed at deprescribing sulphonylureas/insulin in older people with T2D who are treated in general practice.

**Trial registration:**

ISRCTN Registry, ISRCTN50008265, registered 09 March, 2023.

**Supplementary Information:**

The online version contains supplementary material available at 10.1186/s13063-024-08249-9.

## Background and rationale {6a}

Type 2 diabetes (T2D) is one of the most prevalent chronic diseases in the world. In 2021, the global prevalence of diabetes was ~ 537 million cases with the majority diagnosed as T2D [1]. Specifically, the global number of older patients (aged ≥ 65 years) with T2D is expected to increase from 122 million in 2017 to 253 million in 2045 [2]. One of the primary aims in the treatment of T2D is reducing blood glucose levels to reduce symptoms and even more importantly to prevent microvascular complications such as retinopathy and neuropathy. However, older patients with T2D may benefit less from glucose-lowering medication since high glucose levels are primarily associated with microvascular disease, which develops in the long term [3]. Importantly, it has been shown that older patients with T2D have a higher risk of hypoglycaemic events than younger patients, which is related to the use of sulphonylureas or insulin [4–6]. Severe hypoglycaemic events in older patients increase the risk of cardiovascular events, dementia and death from any cause [5, 7, 8]. Moreover, most hypoglycaemic events in older patients go unnoticed, since the symptoms are often atypical and include falls, transient ischaemia, nausea and unsteadiness [9].

A study conducted in the Netherlands found that 5.7% of all preventable hospital admissions in older patients were caused by hypoglycaemia due to sulphonylureas and/or insulin treatment [10]. Similar results of overtreatment in older patients with T2D have been found in the UK [11]. In addition, most evidence from observational studies also points against a tight glucose control in older patients with T2D particularly when comorbidity is present [12]. These findings argue for a relaxation of glucose management in older patients. In the Netherlands, most patients with T2D are being treated in general practice. To optimise glucose management in older patients (≥ 70 years), the Dutch College of General Practitioners (NHG) adopted a guideline to allow for higher HbA1c levels in this population. These guidelines also consider the time since the onset of diabetes, the presence of frailty and the life expectancy of the patient [13, 14].

Nevertheless, deprescribing glucose-lowering medication in older people with T2D in general practice is not yet widely adopted [15]. Barriers that have previously been identified for reducing medication include a lack of time of healthcare providers, not willing to change medication started by others, no need to change medication in a-symptomatic patients, and a fear of patients to change their medication [16–20]. In addition, evidence that a reduction in glucose medication in older patients with T2D can be safely and cost-effectively implemented is currently lacking [21].

## Objectives {7a}

**Fig. 1:**
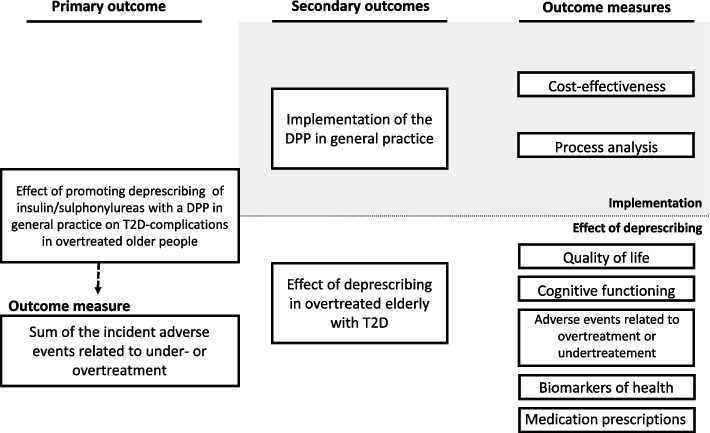
Primary and secondary outcomes of the OMED2-study. DPP: deprescribing programme; T2D: type 2 diabetes; ICPC: International classification of primary care

Therefore, the primary goal of the OMED2 (*Optimization of Medication in Elderly with Diabetes*) study is to investigate the effect of promoting the deprescribing of insulin/sulphonylureas in general practice on T2D-complications in older patients (≥ 70 years) who are overtreated. For this purpose, a deprescribing programme (DPP) will be implemented in general practice that aims to reduce glucose-lowering medication in overtreated older patients, thereby increasing HbA1c levels and reducing hypoglycaemic events. Secondary objectives include assessing the implementation of the DPP in general practice as well as examining the effect of deprescribing insulin/sulphonylureas on health outcomes (Fig. 1).

## Trial design {8}

A parallel-group (1:1) cluster randomised controlled trial with a non-inferiority hypothesis will be performed in general practices in the Netherlands. The study has a hybrid design since it focuses both on the effect and process evaluation of implementing the DPP in general practice as well as on the effect of deprescribing of insulin/sulphonylureas on health outcomes in overtreated older patients with T2D [22] (Fig. 1).

## Methods: participants, interventions and outcomes

### Study setting {9}

The study will be performed in general practices in the Netherlands. The study entails a multicentre trial, with researchers from Amsterdam UMC and Leiden UMC conducting the study.

### Eligibility criteria {10}

**Fig. 2:**
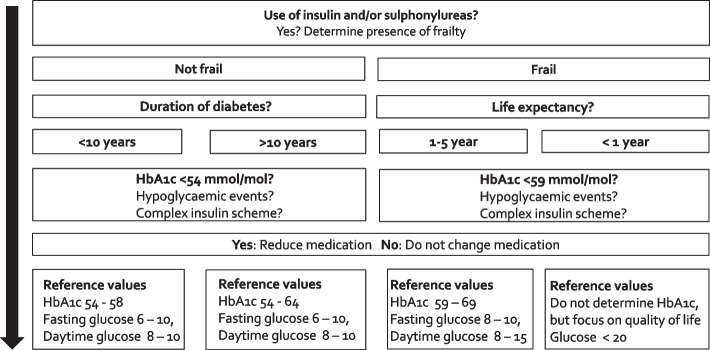
Flow chart for the decision of reduction of glucose-lowering medication. HbA1c-levels in mmol/mol and glucose levels in mmol/l

All general practices that are willing to participate in this study and that provide a signed contract with Amsterdam UMC and a filled-out registration form will be eligible for participation. Older patients (≥ 70 years) with T2D who are potentially overtreated with sulphonylureas and/or insulin will be eligible for the study. Potential overtreatment will be defined as an HbA1c level < 54 mmol/mol. Target levels of HbA1c depend on the presence of frailty, diabetes duration and life expectation (see Fig. 2) [13]. The most recently measured HbA1c level will be used to determine overtreatment. The presence of frailty will be clinically assessed by the general practitioner (GP) and the practice nurse (PN) in the general practice and will be based on the Dutch diabetes guidelines.

### Who will provide informed consent? {26a}

Since the intervention is part of the recommended care in the Dutch diabetes guideline and data from patients are analysed anonymously, no informed consent is needed from the patients for use of regular care data. As such, regular care data from all eligible patients can be used for this study, except for patients who proactively used the so-called opt-out provision and objected sharing their electronic medical record (EMR) data for research purposes. All patients in both the intervention and the control practices who are eligible for study participation will receive a written invitation from the GP, including an informed consent form, to receive questionnaires and to allow the researchers to request medical information from their healthcare provider if needed. Patients who are eligible for the study but are deemed mentally burdened (e.g. due to major life events) or are otherwise deemed ineligible for the invitation to fill in questionnaires will be included in the study analysis but will not be approached for informed consent and further study procedures.

### Additional consent provision for the collection and use of participant data and biological specimens {26b}

For the process analysis, interviews will be conducted with participants and healthcare providers who are enrolled in the DPP intervention and who have given informed consent for this. Participants will give their consent for interviews via the informed consent form and healthcare providers via the contract. With the informed consent, participants and healthcare providers allow that the interviews will be audiotaped and that the data will be used for qualitative data analysis. No biological specimens will be collected within this study.

## Interventions

### Explanation for the choice of comparators {6b}

In order to promote the deprescribing of glucose-lowering medication in general practice, we have developed a DPP and subsequently tested it in a pilot study. Our pilot study (not yet published) showed qualitatively the barriers and facilitators for the implementation of the DPP. Importantly, these results highlighted that the DPP needs to be embedded in the standard care of general practice and should have a minimal time investment from GP and PN. In this study, we will use the adjusted DPP using insights from the pilot study. The comparator will be usual care in the control practices.

### Intervention description {11a}

Practices enrolled in the intervention group will receive education and support for deprescribing insulin/sulphonylureas.

#### Education

**Fig. 3:**
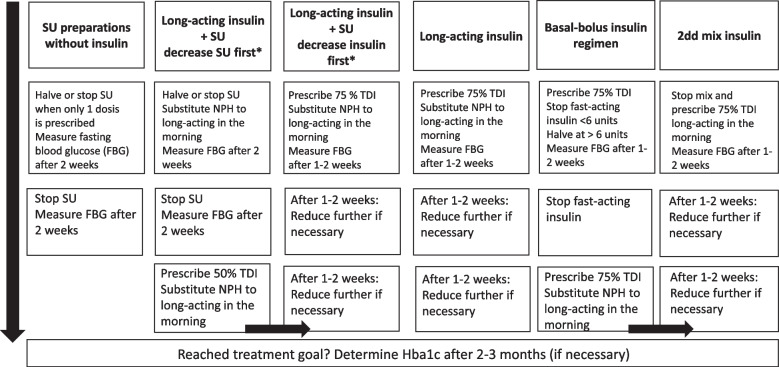
Decision tree for gradually deprescribing sulphonylureas and/or insulin. dd: daily dose; TDI: total daily insulin; SU: sulphonylureas; FBG: fasting blood glucose. *If patients use both insulin and sulphonylureas it is generally advised to decrease sulphonylureas first

After general practices are randomised into the intervention, both GPs and PNs will attend a 1.5-h online group training session from a GP who is an expert in the field of T2D. During this training, information will be provided on the risks involved in too strict glucose management in older patients with T2D and on the HbA1c criteria that can be used to identify overtreated patients. Moreover, healthcare providers will be introduced to a flow diagram which they can use to identify overtreated patients (Fig. 2) as well as a decision tree to select the most optimal course of treatment (Fig. 3). In addition, healthcare providers will be introduced to the Mini-Cog^©^, a test that will be recommended to use for early identification of cognitive dysfunction [23]. The Mini-Cog is explained in more detail in “Outcomes {12}”. Additional training components will include estimating life expectancy and level of frailty as well as conversation and shared decision making strategies to discuss deprescribing with an older patient with T2D. The training will also highlight the option to request a medication review from the local pharmacy or the research team. Training material will be evaluated throughout the study and optimised if necessary to maximise knowledge transfer. In addition to the training, intervention practices will also receive educational materials.

After the training, PNs will be invited to digital supervised peer-to-peer sessions which occur on a monthly basis. During these sessions PNs can share their experiences with the DPP and will discuss a theoretical deprescribing case study. There will be four different peer-to-peer sessions that PNs can attend to.

#### Support

Throughout the study, participating healthcare providers will be able to consult the expert team of the study, consisting of a GP with expertise in T2D, a gerontologist, an endocrinologist, and a pharmacologist. Furthermore, general practices will be visited on two occasions by trained research assistants (medical students) to receive support with the identification of patients that may be eligible for deprescribing. These patients will be identified with a selection tool, as described in Recruitment {15}, which generates a list of possibly overtreated patients. Research assistants will go through the EMR of the general practice to assess which of the patients on the list they find eligible for deprescribing. Subsequently, they will discuss the possibly eligible patients with the healthcare provider and will give advice about deprescribing. Additionally, during the second visit, research assistants will follow up on the deprescribing of patients included during the first visit and give further advice if necessary. The practice visits also give an opportunity for healthcare providers to ask questions about deprescribing to the research assistants who can, if necessary, also relay the question to the study’s expert team. Finally, throughout the study healthcare providers will have the opportunity to request a medication review from the local pharmacy or the research team.

### Criteria for discontinuing or modifying allocated interventions {11b}

Throughout the study, GPs and PNs will remain responsible and make the final decision regarding the medical treatment of the study participants. As such, they are allowed to deviate from the protocol.

### Strategies to improve adherence to interventions {11c}

The GPs and PNs enrolled in the study will receive gift certificates when they attend a schooling or a peer-to-peer session (25–50 euros per hour of attendance for PNs and GPs respectively).

### Relevant concomitant care permitted or prohibited during the trial {11d}

This study does not involve restrictions regarding concomitant care.

### Provisions for post‑trial care {30}

This study does not involve provisions for care after the trial has ended.

### Outcomes {12}

#### Primary outcome measure

The primary outcome is the sum of the incident registered International Classification of Primary Care (ICPC) contact diagnosis codes in the EMR [24, 25] that are related to undertreatment or overtreatment during the 2-year follow-up period of the study. The diagnosis contact codes were identified as being related to under- or overtreatment by means of a Delphi procedure made by four GPs with clinical experience of more than 15 years and expert knowledge on diabetes (see Table 1 for the complete list). The ICPC diagnosis codes of each registered contact will be registered by GPs and PNs continuously throughout the study since this ICPC coding is performed in the Netherlands as part of usual care. To compare the 2-year incidence of ICPCs related to undertreatment and overtreatment, the median sum of ICPC codes and interquartile range (IQR) in 2 years will be calculated for the DPP intervention and the control (usual care) arm.

**Table 1:** ICPC codes for overtreatment and undertreatment as determined with a Delphi procedure

ICPC codes	Over treatment Description	ICPC codes	Under treatment Description
A04.00	Weakness/tiredness general	A04.00	Weakness/tiredness general
A05.00	Feeling ill	A05.00	Feeling ill
A06.00	Fainting/syncope	A06.00	Fainting/syncope
A13.00	Concern about/fear of medical treatment	A09.00	Sweating problem
A29.00	General symptom/complaint other	A29.00	General symptom/complaint other
A85.00	Adverse effect medical agent	D09	Nausea
D09.00	Nausea	D10.00	Vomiting
D10.00	Vomiting	D11.00	Diarrhoea
K04.00	Palpitations/awareness of heart	D20.00	Mouth/tongue/lip symptom/complaint
K79.02	Ventricular tachycardia	F83	Retinopathy
L72.00 – L76	Fractures: radius/ulna	K01.00	Heart pain
L77.00 /L78	Sprain/strain of ankle or knee	K02.00	Pressure/tightness of heart
L78.00	Sprain/strain of knee	K74	Angina pectoris
L80.00	Dislocation/subluxation	N94	Neuropathy
L81.00	Injury musculoskeletal NOS	S84.00	Impetigo
N17	Vertigo/dizziness	T08.00	Weight loss
N79.00	Concussion	U01.00	Dysuria/painful urination
N80.00	Head injury other	U98.00	Abnormal urine test NOS
P01.00	Feeling anxious/nervous/tense	U04.00	Incontinence
P03.00	Feeling depressed	U71	Cystitis/urinary infection
P04.00	Feeling/behaving irritable/angry	X72.00	Genital candidiasis female
P20.00	Memory disturbance	X84.00	Vaginitis/vulvitis NOS
S16.00	Bruise/contusion	Y07.00	Impotence NOS
		P20	Memory disturbance
T87.00	Hypoglycaemia		
U04.00	Incontinence urine		
A80 L72-L76	Fall All Fractures		

#### Secondary outcome measures

The secondary outcome measures of this study are quality of life, cognitive functioning, adverse events related to overtreatment or undertreatment, biomarkers of health, amount and dose of blood glucose-lowering medication prescribed, cost-effectiveness and outcomes of a process analysis. The Standard Protocol Items Recommendations for Interventional Trials (SPIRIT) diagram in Table 2 presents an overview of the timeline of these measurements.

**Table 2:**
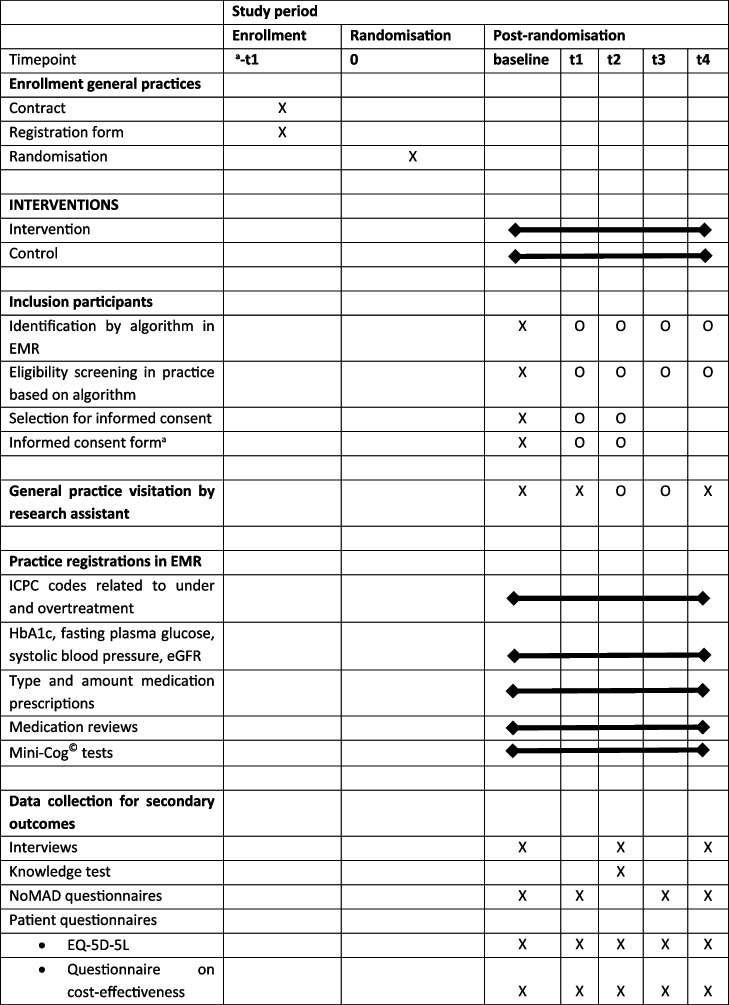
Standard Protocol Items Recommendations for Interventional Trials (SPIRIT) diagram of the OMED2-study [26]

*t* Time in slots of 6 months, *O* Optional inclusion when follow-up does not exceed study duration (for study inclusion) or when visitation is deemed desirable by general practices, *EMR* Electronic medical records, *ICPC* International classification of general practice, *NoMAD* Normalization Measure Development, *EQ-5D-5L* EuroQol five-level

^a^Informed consent is only required for secondary outcome measurements

##### Quality of life

The quality of life of participants will be measured using the validated EuroQol five-level (EQ5D-5L) questionnaire. The EQ5D-5L measures five dimensions of health, i.e. mobility, self-care, usual activities, pain and discomfort, anxiety and depression. Participants rate each of these dimensions on a five-level scale (from no problems to extreme problems). The scores of patients will be translated into a utility score, following a validated Dutch scoring formula [27]. In addition, the EQ5D-5L also consists of the measurement of overall health by means of a visual analogue scale (VAS) with the extreme ends reflecting the worst health state you can imagine (VAS score 0) and the best health state you can imagine (VAS score 100) [28]. The change in quality of life from baseline until end of follow-up will be estimated by comparing EQ5D-5L utility score and EQ5D-5L VAS scores between baseline and end of follow-up. Differences in changes scores (median and IQR) between the two study arms will be compared.

##### Cognitive functioning

Studies have shown that a decline in cognitive function is often accompanied by hypoglycaemic events in older patients [8, 29]. To assess cognitive decline in older patients, GPs and PNs in the intervention arm will be encouraged to use the Mini-Cog^©^ measurement [23]. The Mini-Cog^©^ will only be performed if the GP or PN suspects a cognitive decline in a patient. The Mini-Cog^©^ is a 3-min instrument which consists of a three item recall test and a scored clock drawing test. Patients will be provided with three words, which they will need to repeat to the healthcare provider and will be instructed to remember. Subsequently, patients will be instructed to draw a clock on which the time is 10 min past eleven. Finally, they will be asked to recall the words they were instructed to remember before drawing the clock. Separate scores will be calculated for performance in the item recall test and clock drawing test. Adding these scores will give an indication for the likelihood of dementia. The median change (± interquartile range) in Mini-Cog score between before deprescribing and after deprescribing will be calculated for patients in the intervention arm.

##### Adverse events related to overtreatment or undertreatment

The number of adverse events related to overtreatment and undertreatment will also be assessed separately in a similar way as the primary outcome and using the same list of ICPCs (Table 1). Method of aggregation and main timepoints of comparison will be similar to the primary outcome.

##### Biomarkers of health

From the EMR, HbA1c, fasting plasma glucose, systolic blood pressure and eGFR will be obtained. Measurements of these parameters are part of usual care and are registered by GPs and PNs. According to the Dutch GP diabetes guideline, all these measures should be registered at least once a year. Of primary importance will be to assess the change in biomarkers during the 2-year follow-up period in the DPP intervention arm vs the control arm. The mean change ± standard deviation will be presented to compare intervention with control arm.

##### Amount and dose of blood glucose-lowering medication prescribed

All blood glucose-lowering medication prescriptions will be registered in the EMR throughout the 2-year follow-up period by GPs and PNs as part of usual care. These will be used to calculate the amount of blood glucose-lowering medication prescribed, which will be based on the daily dosages prescribed. Total daily medication dosages will be presented as mean ± standard deviation for intervention and control arm to allow for comparison.

##### Cost-effectiveness

The cost-effectiveness analysis (CEA) of the DPP will be based on the questionnaires of the patients who have given informed consent to receive questionnaires. The questionnaire assesses the nature and frequency of different types of formal (healthcare) and informal care received during the previous 3 months and will be sent at the start of the study and subsequently after every 6 months until the 2-year follow-up period has been reached. The questions which will be used to assess cost-effectiveness are depicted in Additional file 1. All units of healthcare use will be valued using Dutch references prices for healthcare (e.g. GP visits, drugs, inpatient and outpatient visits) and non-healthcare use (e.g. hours of informal care received) [30]. Total costs of the 2-year follow-up period will be added to arrive at individual cost estimates for all participants. Mean costs will be calculated and compared between groups. Costs for the second year will be discounted using a discount rate as advised in Dutch guidelines for health economic evaluation [31].

The outcome measure for the cost-effectiveness analysis is cost per QALY gained. The number of QALYs for every participant and the mean number of QALYs for both groups will be estimated using the EQ5D-5L questionnaire (see above under quality of life). The outcomes of the EQ5D-5L utility scores will be used to calculate QALYs. Quality of life will be measured at baseline and every 6 months until the 2-year follow-up period has been reached (Table 2). QALYs will be estimated using an area under the curve approach. The mean of two consecutive 6-month utility scores will be corrected by a factor 0.5 (reflecting a half year period) and utility scores of four consecutive half year periods will be added to calculate the total number of QALYs realised during a 2-year follow-up period. Cost differences between groups will be divided by QALY differences between groups to estimate the cost per QALY gained. Bootstrap analysis will be performed to account for uncertainty of the estimates. A cost-effectiveness acceptability curve (CEAC) will be drawn based on 5000 bootstraps. The CEAC will show the probability that the intervention is cost-effective at different threshold levels for cost-effectiveness, e.g. the Dutch willingness to pay thresholds of €20,000, €50,0000, and €80,000 per QALY.

##### Process analysis

A process analysis will provide insight into the extent to which the DPP has been performed according to the study protocol and identifies barriers and facilitators for the execution of the DPP. Furthermore, the implementation of the DPP in general practice will be evaluated by means of the Reach, Efficacy–Adoption, Implementation, Maintenance (RE-AIM) model [32] and the Extended Normalisation Process Theory (ENPT) [33]. Reach of the intervention will be expressed as the absolute and relative number of participating older patients with T2D. It will also be assessed how representative the research population is for the total group of older patients with T2D. Efficacy will be the impact of the intervention on outcomes such as quality of life. Adoption is the absolute number of included patients for whom deprescribing is initiated and who complete the total 2-year follow-up period. Implementation refers to the degree of consistency of the execution of the different components of the DPP. Maintenance cannot be addressed in this study, as it is outside the scope of this study to investigate the institutionalisation and long-term effect on the individual level. The ENPT theory consists of four core elements: potential, capacity, capability and contribution. Combined, these elements reflect the stakeholders’ effort to normalise a complex intervention. To quantitatively address components of RE-AIM and ENPT, healthcare providers will be instructed to fill out the Normalization Measure Development (NoMAD) questionnaire [34]. The questionnaire consists of 20 statements about the implementation of the DPP. Healthcare providers will indicate how much they agree with each statement. The NoMAD questionnaire will be sent to GPs and PNs after the training session with the T2D expert (at the start of the study) and every 6 months thereafter until the end of the 2-year follow-up period has been reached. Of primary importance will be the change in NoMAD score between the score of the first NoMAD questionnaire and the score of the last NoMAD questionnaire (after 2 years of follow-up). Median ± interquartile range will be calculated to compare the score of the first NoMAD questionnaire with the score of the last NoMAD questionnaire. In addition, in the intervention arm, interviews with healthcare providers and patients will also be performed for qualitative assessment of components of RE-AIM and ENPT. We aim to conduct a total of 20 interviews with patients and healthcare providers combined throughout the study. For these interviews, we will develop a topic list based on ENPT for healthcare providers and based on the systematic review of Reeve et al. [16] for the patients. The topic list will be adjusted and optimised during the period that the interviews are being conducted. Both the total number of interviews and the content will depend on the richness of the interviews and the information obtained. Patient sampling will be done purposeful to select a diverse population with different attitudes towards deprescribing. Also, we shall analyse the free text of the EMR using the same frameworks, after erasing all potential identifying information. Additionally, 1 year after the training, the healthcare providers of both the intervention and control group will be given an online multiple-choice test about deprescribing in older patients with T2D. The purpose of this test is to assess how much knowledge from the DPP is still present in the healthcare providers and to compare their knowledge with healthcare providers in the control practices. The topics that will be addressed in the test have been covered during the training session of the intervention group. From the test results, the percentage of correctly answered questions will be calculated. The median ± standard deviation of these percentages will be reported separately for the intervention and control arm to allow for comparison.

### Sample size {14}

Previous analysis of the EMR extractions of 31 practices of the Academic GP Network Amsterdam identified 310 patients that were suitable for the DPP programme of our study. A previous study showed that 55% of the identified patients had an indication for the DPP. In the identified DPP patients in the EMR, we found 2.4 registered ICPC codes that formed part of our primary outcome measure in an average follow-up period of 1.8 years. Using a non-inferiority design with a non-inferiority ratio of 1.1 with an absolute difference of 0.2 codes, a non-inferiority limit of < 0.1, a power of 0.8, a significance level of 0.05 and taking into account the multilevel design of the study and a loss to follow-up of 10%, we set our sample size to 406 patients and 74 participating practices. We used the NCSS Statistical Software Package (NCSS, Utah, USA) for the power calculation. This package is designed to calculate sample size using a Poisson distribution [35]. This package does not take clustering into account. Therefore, we applied a correction factor for a multilevel design [36]. An intra-class correlation coefficient (ICC) of 0.12 was assumed. Both the non-inferiority limit and the estimation of the ICC were set based on the judgement of the research group. In the previously mentioned (not yet published) pilot study in 10 GP practices with a follow-up of 6 months, an average of 5.5 patient per practices were selected for deprescribing. Since these practices were highly motivated practices with ample experience in performing healthcare research, we increased the number of participating practices with an additional 15% to 85 practices and one extra practice to reach an even sample size of 86.

### Recruitment {15}

General practices will be recruited by the Department of General Practice of Amsterdam UMC and by the Department of Public Health and Primary Care of Leiden University. General practices will be recruited via care groups and the networks of the investigators. Patients will be recruited via the general practice in which they are registered in the period March 2023 to March 2024. To recruit older patients that are eligible for participation in the study, a selection tool will be used. The selection tool consists of an algorithm that uses birth date and care registration data from the EMR of the general practice to generate a list of patients that may be eligible for deprescribing (Additional file 2). Research assistants will visit the general practice to assess which patients from the list are indeed eligible for participation. For analytical purposes, a note will be made in the EMR to follow the patients that were identified by the algorithm. In addition, in consultation with the healthcare provider, research assistants will sent out questionnaires and ICFs which are used for the measurement of secondary outcomes. For the control practices, patient identification of patients eligible for deprescribing will not be shared with the healthcare providers. See Intervention description {11a} for further details on the practice visits in the intervention practices.

A list of possibly eligible patients will be generated at the start of the study. A new list with potential patients will be generated after 6 months, permitted that the 2-year follow-up for each patient does not exceed the study duration (Table 2).

## Assignment of interventions: allocation

### Sequence generation {16a}

Randomisation will be performed on the level of the GPs and PNs. Periodically, all eligible practices that are not yet randomised and have signed the contract will be grouped into two groups of comparable patient size. The practices that share PNs will be put in the same group. Subsequently, the two strata will be randomised by using a digital die.

### Concealment mechanism {16b}

Randomisation will only be done after the practice has signed the contract with Amsterdam UMC to perform the study. At least two researchers will perform randomisation to ensure that the randomisation process is not influenced by an individual researcher (i.e. to limit the possibility of selection bias).

### Implementation {16c}

Healthcare providers will enrol participants into the study. This process will be guided by the selection tool of the DPP and by the administrative support of the research assistant who will be visiting the general practices.

## Assignment of interventions: Blinding

### Who will be blinded {17a}

General practices will receive education on the interventions and therefore these practices cannot be blinded. Patients will be informed that the university is studying medication use in T2D, but are not made aware of the specifics of the study, i.e. the DPP. Due to the nature of the study, blinding of the data analysis is not possible. Data is collected from the EMR, which contains patient-specific information including deprescribing of medication. To preserve the integrity of the study results, analyses will be discussed during research group meetings.

### Procedure for unblinding if needed {17b}

Since the study is open, no unblinding procedures are needed.

## Data collection and management

### Plans for assessment and collection of outcomes {18a}

Extraction of the EMR of general practices will be done by either the Academic GP Research Network of Amsterdam UMC (Academisch Netwerk Huisartsengeneeskunde Amsterdam (ANHA)) or the Instituut voor Zorgoptimalisatie (INSZO). These institutions act as Trusted Third Parties and will make the data available to the researchers in a protected digital environment (myDRE), which does not contain personal information of the patients. These data include the type of medication prescribed, biomarkers of health (i.e. HbA1c, fasting plasma glucose, systolic blood pressure and eGFR), lists of ICPC episodes and free text noted by the GP or PN. The data from the medical file will be used to assess whether patients were indeed eligible for deprescribing and to follow the process of implementation. The free text will first pass an anonymization tool, since free text in the medical file can contain patient identifying information. Additionally, general information per practice, i.e. total number of patients registered and number of patients with T2D at the start of the study, will also be available. Data extractions from the EMR will be performed at 12, 18 and 32 months after the first practice visit, with the last date of data extraction not exceeding October 2025. Patients who have given informed consent to receive questionnaires will receive a code in their medical file so that medical file data can be linked to the questionnaires and the interviews. These patients have also specifically given informed consent for the sharing of medical data with the research group.

The NoMAD questionnaires will be sent to the healthcare providers via the data management platform Castor EDC as electronic forms which are directly linked to the data management system [37]. Patient questionnaires will be sent to patients via regular mail and data received will be entered manually into the Castor EDC database by research assistants. The knowledge test that healthcare providers receive as part of their education will also be sent digitally via Castor.

### Plans to promote participant retention and complete follow‑up {18b}

Practices receive a visit by a research assistant at the start of their participation in the study, after 6 months and at the end of the study (if necessary to collect additional information). During the first two visits, all issues concerning the DPP and study-specific questions will be discussed, and the practice will be helped with the administrative procedures. In the general practice, a list of all included patients will be stored and the research assistant will systematically discuss these patients with the PN. The research assistant will either help by providing additional information and explanation or by stimulating contact with the expert team of the research group. During all practice visits, anonymized notes are made by the research assistants concerning the problems that the healthcare providers encounter. These notes will be analysed for implementation purposes and to improve study materials.

Healthcare providers will be reminded once by the data management platform Castor EDC if no reply has been received after 7 days. In case of repetitive non-reply or technical difficulties, the NoMAD questionnaire will be sent to the healthcare provider in PDF via e-mail. Participants will receive a phone call from one of the research members in case of not returning or incompletely filling out the patient questionnaires.

### Data management {19}

Personal information about patients selected from the EMR by the selection tool will remain at the general practices. Prior to data extraction, an extraction plan will be made which will specify all the data relevant to the study. Based on this plan, data will be extracted from the EMR by ANHA and INSZO in a coded manner, so that the researchers have no personal information of the patients except for the patients that have given informed consent.

### Confidentiality {27}

Confidentiality agreements will be signed during each visit of the research assistant to the general practices. An additional confidentiality agreement will be signed for use of the data extraction from the EMR in the protected digital myDRE environment. Members of the research team will need to log into the myDRE environment in order to access the EMR data. Login codes are also required for access to Castor EDC. Participant identifiable data and audiotapes will be stored separately in a protected digital environment or in a locked closet. Research data will be stored for 15 years.

### Data access {29}

Any data required to support the protocol can be supplied upon reasonable request.

### Plans for collection, laboratory evaluations and storage of biological specimens for genetic or molecular analysis in this trial/future use {33}

No biological specimens will be collected or stored in this study.

## Statistical methods

### Statistical methods for primary and secondary outcomes {20a}

The primary outcome of this study will be the sum of hypo- and hyperglycaemic-related adverse events, which will be analysed using a generalised linear model with the DPP intervention as the main determinant and number of events as the dependent variable. Cluster-specific random effects will be included in the regression model. Secondary outcomes consist mainly of continuous outcome variables and therefore these will be analysed using multilevel linear mixed models. Both an intention-to-treat as well as an analysis that only includes patients for whom deprescribing of insulin and/or sulphonylureas was initiated will be performed. The intention-to-treat analysis will include all patients who are eligible for deprescribing, regardless if actual deprescribing. A *p*-value < 0.05 will be considered significant. Patient utility scores, reflecting the valuation of quality of life of patients, will be calculated from the EQ5D-5L questionnaires, following a published Dutch valuation scoring formula [27]. By combining utility scores and follow-up time (until end of study or dying), QALYs are estimated on a patient level. This will be done using interpolation of scores between different measurements (area under the curve approach). Average number of QALYs in the two patient groups will be compared. Costs will be based on the data provided by patients and will be estimated from a societal perspective, implying that both healthcare costs and patient- and family costs are included. Given the advanced age of patients, productivity costs are ignored in this study. For all patients, the number of units of different types of costs is counted during follow-up. The number of units is multiplied by reference costs per type of healthcare, as published by the National Healthcare Institute [30]. For all patients, total costs over the follow-up time are estimated and average costs of intervention and control patients will be compared. A cost-utility analysis (CUA) will be performed by dividing the average cost differences between intervention and control patients by the average differences in QALYs. Uncertainty analysis will be done using bootstrapping with a minimum of 5000 draws. Results of CEA will be presented with cost-effectiveness acceptability curves, i.e. the probability that the intervention is cost-effective compared to usual care at accepted levels of societal willingness to pay for a QALY. Level of implementation will be evaluated using the ENPT and the RE-AIM method.

### Interim analysis {21b}

No interim analysis will be performed regarding the safety of the DPP programme.

### Methods for additional analyses (e.g. subgroup analyses) {20b}

Analysis will be done based on intention to treat to test the DPP. We will perform a secondary analysis to study the effect of the medication reduction in insulin and/or sulphonylurea on our primary and secondary outcomes. It is likely that some patients will again receive more medication after deprescribing or for whom deprescribing will not be initiated at all. Subgroups will be made to compare those patients with the ones whom fully completed deprescribing as indicated in the decision tree.

### Methods in analysis to handle protocol non‑adherence and any statistical methods to handle missing data {20c}

Protocol non-adherence will be studied during the process analysis as described in Outcomes {12}. Intention-to-treat analyses will be performed which will include all patients who are eligible for deprescribing, as determined during the practice visits. This analysis will include patients with and without a reduction in glucose-lowering medication in intervention and control group. We have selected analysis techniques that are relatively insensitive to missing data, and no imputation will be performed.

### Plans to give access to the full protocol, participant‑level data and statistical code {31c}

The study protocol and statistical codes used can be made available upon reasonable request. Patient data will only be shared with external parties if patients gave permission for this on the informed consent form.

## Oversight and monitoring

### Composition of the coordinating centre and trial steering committee {5d}

The coordinating study centre is the Department of General Practice, Amsterdam UMC, the Netherlands. The trial steering committee consists of members of both Amsterdam UMC and Leiden UMC. Both study centres will perform study planning, recruitment and study monitoring, whereas the coordinating centre will be responsible for randomisation, data registration, data management and biostatistics. Monthly meetings will be scheduled with the trial steering committee. In addition, a patient advisory group has been established for this study which will meet twice a year with the trial steering committee. Finally, throughout the study, meetings will be planned with all stakeholders involved in the study at critical time points and at least once a year to discuss study progress and study challenges. This group consists of experts in the field of T2D, endocrinology, health economics, pharmacology and geriatrics. The principal investigator together with the project leader are the main responsible persons for trial oversight and making all relevant decisions within the trial.

### Composition of the data monitoring committee, its role and reporting structure {21a}

The expert trial steering committee of the OMED2-trial appraised the trial as low risk; therefore, the investigators did not choose to make use of a data safety and monitoring committee.

### Adverse event reporting and harms {22}

The medical ethical committee of the Amsterdam UMC, location Vrije Universiteit, has deemed that the study does not fall under the Medical Research Involving Human Subjects act (WMO). Therefore, the reporting of adverse events will conform to usual care legislation in which several Dutch laws apply: Dutch Medical Treatment Contracts act (referred to as WGBO) and the Healthcare Quality, Complaints and Disputes Act (referred to as Wkkgz). The rapportage means that the events will have to be noted in the medical file, that a separate file is being kept and that serious adverse events are reported to the authorities. Since the study relies on care registration data, adverse events will be collected non-systematically as ICPC codes (see also Outcomes {12}).

### Frequency and plans for auditing trial conduct {23}

No monitoring visits will be planned for this trial.

### Plans for communicating important protocol amendments to relevant parties (e.g. trial participants, ethical committees) {25}

Important protocol amendments will be communicated to the medical ethical committee and reported in study publications. Protocol amendments will also be communicated to all researchers involved in the study.

### Dissemination plans {31a}

We aim to publish study effectiveness, process analysis and cost-effectiveness of the intervention in peer-reviewed journals. Relevant study results and materials will also be communicated with healthcare providers.

### Authorship eligibility guidelines and any intended use of professional writers {31b}

For future trial publications, authorship will be determined on the level of involvement of the researcher in the manuscript. Members of the trial steering committee will be able to indicate on the statistical analysis plan which research questions they want to be involved in.

## Discussion

This paper depicts the background and study design of the OMED2-study, a study to evaluate the safety of a programme aimed to reduce insulin and/or sulphonylureas in older patients with T2D that are overtreated by healthcare providers in general practice. It is hypothesised that a reduction in blood glucose-lowering medication in this population can be safely implemented, which will be reflected by a similar incidence of diabetes complications between the deprescribing (intervention) group and control group (usual care) during the 2-year follow-up period of the study. In addition, this study will provide insight into the implementation of the deprescribing programme (DPP) in the general practice by means of a process analysis.

This complex intervention study is expected to encounter challenges. It is possible that general practices that are randomised into the intervention group could exchange their knowledge on deprescribing with healthcare providers in the control group. This transfer of knowledge could result in cross-contamination between the intervention and control group, thereby diluting the intervention effect. To reduce this risk, the control group will be offered support with the selection of patients with T2D and a very high risk of cardiovascular complications who are younger than 70 years of age, thereby possibly eligible for starting sodium-glucose transporter 2 inhibitors (SGLT2i) or glucagon-like peptide-1 (GLP1) medication. This (control) intervention includes education and a selection tool and will not interfere with the DPP intervention since the interventions target different groups of patients, i.e. young (< 70 years, control intervention) and old (≥ 70 years, DPP programme).

In addition, randomisation will be on the level of the general practice and PN. This form of randomisation has been chosen from a practical point of view, since GPs within the same general practice often share PNs. Thus, this form of randomisation prevents that PNs who work for different practices are randomised in the intervention in one practice and in the control group in the other. It is, however, likely that patients who visit the same general practice or are treated by the same PN will receive similar care which could affect their frequency of diabetes complications. Since we expect clustering of observations both at the level of the practice as at the level of the patient, variables of cluster-specific random effects will be included in the statistical analysis [38].

From our previous pilot study (not yet published), it became clear that the deprescribing of sulphonylureas and/or insulin in older patients is not easily implemented in the general practice. The main struggle of healthcare providers was that new skills in selecting patients using the selection tool as well as shared decision making needed to be acquired for deprescribing. In line with this observation, results from our pilot study showed that a prerequisite for the successful implementation of the DPP programme in general practice is that it can be easily integrated in the usual care and does not ask for research-related activities of the healthcare providers. As such, to support practices, research assistants will visit general practices to help out with the additional administrative burden of performing research. During the pilot it also became apparent that PNs have difficulties in starting up new strategies; the external research assistant who is familiar with the new approach was identified as an important asset. Therefore, we implemented two definitive practice visits by the research assistant in the programme with a third extra one if deemed useful by the practice. The research assistants will help the PN to select eligible patients and we will evaluate whether all innovations of the programme normalise to usual care over time.

Finally, for a successful assessment of the safety of the DPP, the study is dependent on care registration data in the EMR. Previously, it has been indicated that differences exist between general practices in reporting of adverse events in the EMR [39]. Although the research assistants that will visit the general practices can also provide feedback on recording health events in the EMR, this study does not entail an optimisation of the use of the EMR. Since the OMED2-study is a randomised controlled trial it is anticipated that variation in reporting of adverse events will occur in general practices in the intervention group as well as in practices in the control group, which will limit the influence of inconsistent reporting on the study outcomes.

The RCT design is one of the strengths of the OMED2-study. Indeed, most previous studies that investigated the relationship between HbA1c level and health outcomes in older people with T2D were observational studies [40–43]. There are RCTs available that did examine glucose management in older people, but these studies were directed at comparing tightly regulated glucose levels with usual care [44–48]. These RCTs indicated that tightly regulated glucose levels may not be more beneficial for older people [44–48], but may actually increase the risk of hypoglycaemia [46, 47]. Therefore, the OMED2-study will be one of the first RCTs to examine whether relaxation of the glucose regime can be safely implemented in older people with T2D.

To investigate this, the OMED2-study will collect (anonymized) routine care data for the primary outcome measures. This way of data collection negates the necessity to obtain informed consent to measure the primary outcome, and therefore all patients that are overtreated with insulin and/or sulphonylureas are included in the main analysis. Thus, even patient groups that are usually excluded from intervention studies (e.g. due to language barriers or cognitive dysfunction) can also be included. As a consequence, results of this study are minimally influenced by selection bias and the study has a high external validity [49].

If the OMED2-study shows that deprescribing of blood glucose-lowering medication can be safely implemented, this could promote more healthcare practitioners in reducing glucose-lowering medication in older adults with T2D and the cost-effectiveness of our program. Importantly, this study will provide insight into the barriers and facilitators that are faced with when implementing a currently existing guideline into the general practice. It is anticipated that these results will also give insight into the implementation of other guidelines/alterations to the standard practice in the general practice.

## Trial status

Protocol version 1, 31/01/2024

Start recruitment: 13/03/2023

Anticipated end of recruitment: 13/03/2024

Anticipated end date of study: 01/07/2026

## Supplementary Information

Supplementary Material 1.

Supplementary Material 2.

Supplementary Material 3.

Supplementary Material 4.

Supplementary Material 5.

## Data Availability

Data and material can be made available upon reasonable request and if consent has been provided for this. See also section 31c.
