# Supplementary material for: A deprescribing programme aimed to optimise blood glucose-lowering medication in older people with type 2 diabetes mellitus, the OMED2-study: the study protocol for a randomised controlled trial
Source: Trials. 2024 Jul 25;25:505. doi: 10.1186/s13063-024-08249-9 (PMC11271055; doi:10.1186/s13063-024-08249-9)
Supplement: Supplementary file 1 — Supplementary Material 1. [file 13063_2024_8249_MOESM1_ESM.pdf]

Healthcare costs questionnaire

Please find the original version (in Dutch) below

Question 6. How often have you visited the general practice center in the past 3 months? General practice center refers to the general practice that you only visit in the evening, night and during the weekend.

- ☐ Not once
- ☐ …. times

Question 7. How often in the last 3 months have you visited the emergency first aid of a hospital? This refers to the first aid in the hospital

- ☐ Not once
- ☐ …. times

Question 8. Have you been admitted to the hospital in the last 3 months? With admitted we mean that you needed to stay overnight. For example, you needed to stay at the hospital due to surgery.

- ☐ No
- ☐ Yes

Did you answer “Yes”? You can continue to answer question 9 and 10. If not, continue with question 11.

Question 9. How often have you been admitted to the hospital in the last 3 months?

…… times in the last 3 months

Question 10. How long have you been in the hospital in the past 3 months?

Have you been admitted to the hospital more than once in the past 3 months? Then you can add up all days

…… days in total in the past 3 months

Question 11. Have you had help of the homecare in the last 3 months?

☐ No

☐ Yes

Have you answered “Yes”? Continue to answer questions 12 to 14. If not, continue to answer question 15.

Question 12. What kind of homecare have you received in the last 3 months?

You can tick more than 1 box.

☐ Help with domestic chores

e.g.: vacuuming, making the bed, grocery shopping

☐ Self-care

e.g.: help with showering or to get dressed

☐ Nursing

e.g.: putting on bandage, giving medication, measuring blood pressure

Question 13. How many weeks have you received this homecare? Add up all weeks in the last 3 months. Please note: a period of 3 months contains <u>13 weeks</u>.

Help with domestic chores: … weeks in the past 3 months

Self-care : … weeks in the past 3 months

Nursing: … weeks in the past 3 months

Question 14. How many hours of homecare did you receive in these weeks on average?

Help with domestic chores: on average … hour per week

Self-care: on average … hour per week

Nursing: on average … hour per week

Question 15. How often have you been to a daycare in the last 3 months?

☐ Not once

☐ …. times

Thank you very much for answering the questions above. Are you tired and would you like to stop taking the questionnaire? That is allowed.

If you have sufficient energy left to answer the next 7 questions as well we would appreciate that very much.

Question 16. We asked in the questions above if you have been admitted to the hospital. Did you need to stay overnight in another institution in the last 3 months because of your health? For example in a residential/care center, nursing home or a rehabilitation center

- ☐ No
- ☐ Yes

Did you answer “Yes”? Continue to answer questions 17 and 18. If not, proceed with question 19.

Question 17. What kind of institution were you in? You can tick more than 1 box.

- ☐ Residential/care center
- ☐ Nursing home
- ☐ Rehabilitation center
- ☐ Another institution, namely ……………………………………

…………………………………………………………………………

Question 18. For how long have you been in this institution? Did you tick more than one box at question 17? Please indicate below for every institution how long you have been there.

Did you go to an institution more than once in the past 3 months? Add up all days.

In the residential/care center: …… dagen in de afgelopen 3 maanden

In the nursing home: …… dagen in de afgelopen 3 maanden

In the rehabilitation center: …… dagen in de afgelopen 3 maanden

In the other institution: …… dagen in de afgelopen 3 maanden

Question 19. Did you receive in the last 3 months help of a relative or an acquaintance due to your physical or mental problems?

- ☐ No
- ☐ Yes

Did you answer “Yes”? Continue to answer questions 20 to 22.

If not, proceed to the end of the questionnaire.

Question 20. What kind of help of relatives or acquaintances did you receive in the past 3 months? You can tick more than 1 box.

- ☐ Help with domestic chores

  e.g.: vacuuming, making the bed, grocery shopping, preparing food and drinks, taking care of the children

- ☐ Self-care

  e.g.: help with showering or getting dressed, help with eating and drinking or giving medication

☐ Practical help

e.g.: support with walking, taking excursions or visits to acquaintances, visits to the general practitioner or hospital, arranging for help or taking care of financial business

Question 21. How many weeks did you receive this help? Add up all weeks in the last 3 months. *Please note: a periode of 3 months contains* *<u>13 weeks</u>*

Help with domestic chores:	… weeks in the past 3 months
Self-care:	… weeks in the past 3 months
Practical help:	… weeks in the past 3 months

Question 22. How many hours of help did you receive these weeks on average?

Help with domestic chores:	on average … hour per week
Self-care:	on average … hour per week
Practical help:	on average … hour per week

Vraag 6. Hoe vaak bent u in de afgelopen 3 maanden naar de huisartsenpost gegaan? We bedoelen de huisartsenpost waar u in de avond, nacht en weekend naartoe gaat.

☐ Geen enkele keer

☐ …. keer

Vraag 7. Hoe vaak bent u in de afgelopen 3 maanden op de spoedeisende eerste hulp van een ziekenhuis geweest? We bedoelen de Spoedeisende Hulp in het ziekenhuis. Een andere naam voor spoedeisende eerste hulp is EHBO.

☐ Geen enkele keer

☐ …. keer

Vraag 8. Heeft u in de afgelopen 3 maanden wel eens in het ziekenhuis gelegen? U moest dus blijven slapen. Bijvoorbeeld omdat u geopereerd was en niet meteen naar huis kon.

☐ Nee

☐ Ja

Heeft u “Ja” aangekruist? Beantwoord dan vraag 9 en 10.

Ga anders verder met vraag 11.

Vraag 9. Hoe vaak heeft u in de afgelopen 3 maanden in het ziekenhuis gelegen?

…… keer in de afgelopen 3 maanden

Vraag 10. Hoe lang heeft u in de afgelopen 3 maanden in het ziekenhuis gelegen? Heeft u meer dan 1 keer in het ziekenhuis gelegen in de afgelopen 3 maanden? Tel dan alle dagen bij elkaar op.

…… dagen in totaal in de afgelopen 3 maanden

Vraag 11. Heeft u in de afgelopen 3 maanden hulp van de thuiszorg gehad?

☐ Nee

☐ Ja

Heeft u “Ja” aangekruist? Beantwoord dan vraag 12 tot en met 14. Ga anders verder met vraag 15.

Vraag 12. Wat voor hulp van de thuiszorg heeft u gehad in de afgelopen 3 maanden?

U kunt meer dan 1 hokje aankruisen.

☐ Huishoudelijke hulp *voorbeeld: stofzuigen, bed opmaken, boodschappen doen*

☐ Verzorging van uzelf

voorbeeld: hulp bij douchen of aankleden

☐ Verpleging

voorbeeld: verband omdoen, medicijnen geven, bloeddruk meten

Vraag 13. Hoeveel weken heeft u deze thuiszorg gehad? Tel alle weken in de afgelopen 3 maanden bij elkaar op. *Let op: een periode van 3 maanden telt 13 weken*.

Huishoudelijke hulp: … weken in de afgelopen 3 maanden

Verzorging van uzelf: … weken in de afgelopen 3 maanden

Verpleging: … weken in de afgelopen 3 maanden

Vraag 14. Hoeveel uur thuiszorg kreeg u in deze weken gemiddeld?

Huishoudelijke hulp: gemiddeld … uur in de week

Verzorging van uzelf: gemiddeld … uur in de week

Verpleging: gemiddeld … uur in de week

Vraag 15. Hoe vaak bent u in de afgelopen 3 maanden naar een dagopvang geweest?

- ☐ Geen enkele keer
- ☐ …. keer

Heel fijn dat u de vragen hierboven heeft ingevuld. Bent u moe en wilt u stoppen met de vragenlijst? Dat mag.

Als u nog voldoende energie heeft om de volgende 7 vragen ook te beantwoorden vinden we dat heel fijn.

Vraag 16. Hierboven hebben we u gevraagd of u in het ziekenhuis bent opgenomen. Moest u in de afgelopen 3 maanden ergens anders blijven slapen voor uw gezondheid? Bijvoorbeeld in een woon-/zorgcentrum, verpleeghuis of revalidatiecentrum.

- ☐ Nee
- ☐ Ja

Heeft u “Ja” aangekruist? Beantwoord dan vraag 17 en 18.

Ga anders verder met vraag 19.

Vraag 17. Wat voor instelling was dit? U kunt meer dan 1 hokje aankruisen.

- ☐ Woon-/zorgcentrum
- ☐ Verpleeghuis
- ☐ Revalidatie-instelling
- ☐ Een andere instelling, namelijk ……………………………………

……………………………………………………………………………

Vraag 18. Hoe lang bent u in deze instelling geweest? Heeft u bij vraag 17 meer dan 1 hokje aangekruist? Vul dan hieronder voor iedere instelling in hoe lang u er bent geweest.

Bent u ergens meer dan 1 keer geweest in de afgelopen 3 maanden? Tel dan alle dagen bij elkaar op.

In het woon-/zorgcentrum: …… dagen in de afgelopen 3 maanden

In het verpleeghuis: …… dagen in de afgelopen 3 maanden

In de revalidatie-instelling: …… dagen in de afgelopen 3 maanden

In de andere instelling: …… dagen in de afgelopen 3 maanden

Vraag 19. Heeft u in de afgelopen 3 maanden hulp gekregen van een familielid of een bekende vanwege uw lichamelijke of psychische problemen?

- ☐ Nee
- ☐ Ja

Heeft u “Ja” aangekruist? Beantwoord dan vraag 20 tot en met 22.

Ga anders naar het einde van de vragenlijst.

Vraag 20. Wat voor hulp van familieleden of bekenden heeft u gehad in de afgelopen 3 maanden? U kunt meer dan 1 hokje aankruisen.

- ☐ Huishoudelijke hulp

  voorbeeld: stofzuigen, bed opmaken, boodschappen doen, klaarmaken van eten en drinken, verzorgen van kinderen

- ☐ Verzorging van uzelf

  voorbeeld: hulp bij douchen of aankleden, hulp bij het eten en drinken of het geven van medicijnen

☐ Praktische hulp

voorbeeld: ondersteuning bij wandelen, het maken van uitstapjes of bezoekjes aan bekenden, bezoeken aan de huisarts of het ziekenhuis, het regelen van hulp of het regelen van financiële zaken

Vraag 21. Hoeveel weken heeft u deze hulp gehad? Tel alle weken in de afgelopen 3 maanden bij elkaar op. *Let op: een periode van 3 maanden telt 13 weken*.

Huishoudelijke hulp:	… weken in de afgelopen 3 maanden
Verzorging van uzelf:	… weken in de afgelopen 3 maanden
Praktische hulp:	… weken in de afgelopen 3 maanden

Vraag 22. Hoeveel uur hulp kreeg u in deze weken gemiddeld?

Huishoudelijke hulp:	gemiddeld … uur in de week
Verzorging van uzelf:	gemiddeld … uur in de week
Praktische hulp:	gemiddeld … uur in de week
