# Supplementary material for: A deprescribing programme aimed to optimise blood glucose-lowering medication in older people with type 2 diabetes mellitus, the OMED2-study: the study protocol for a randomised controlled trial
Source: Trials. 2024 Jul 25;25:505. doi: 10.1186/s13063-024-08249-9 (PMC11271055; doi:10.1186/s13063-024-08249-9)
Supplement: Supplementary file 2 — Supplementary Material 2. [file 13063_2024_8249_MOESM2_ESM.pdf]

Criteria used for the algorithm to identify people who may be overtreated.

Inclusion criteria for the identification of potential participants via EMR (active patients).

OMED-OLD:

- Age 70 years or over
- Diabetes diagnosis (ICPC T90.2 or prescription of anti-diabetes medication ATC A10)
- Prescription of SU derivates and/ or insulin (ATC-codes: A10BB, A10BC, A10BD02, A10BD04, A10BD06 (SU) or A10AB, A10AC, A10AD, A10AE, A10AF (insulin))
- Last measurement of Hba1c < 54 mmol/mol
- Hba1c measurement before the last measurement < 54 mmol/mol
