# Supplementary material for: A deprescribing programme aimed to optimise blood glucose-lowering medication in older people with type 2 diabetes mellitus, the OMED2-study: the study protocol for a randomised controlled trial
Source: Trials. 2024 Jul 25;25:505. doi: 10.1186/s13063-024-08249-9 (PMC11271055; doi:10.1186/s13063-024-08249-9)
Supplement: Supplementary file 3 — Supplementary Material 3. [file 13063_2024_8249_MOESM3_ESM.pdf]

1. Overeenkomst voor onderzoek met proefpersonen

OMED2-studie (Niet-WMO)

Partijen

a. Stichting VU medisch centrum, gevestigd te De Boelelaan 1117, 1081 HV te Amsterdam, in deze rechtsgeldig vertegenwoordigd door E. Janssen, directeur bedrijfsvoering divisie X, Amsterdam UMC, hierna te noemen "**VUmc**";

en

b. ……………………………………………………………………………….[praktijknaam], gevestigd te ……………………………………………………………………………[praktijk adres], in deze rechtsgeldig vertegenwoordigd door:

……………………………………… [naam praktijkhouder 1];

……………………………………… [naam praktijkhouder 2];
……………………………………… [naam praktijkhouder 3, enzovoort],
hierna te noemen: "**Deelnemende Praktijk**;

in het hiernavolgende afzonderlijk ook aangeduid als "**Partij"** en gezamenlijk aangeduid als "**Partijen**"

Overwegende dat:

- de afdeling Huisartsgeneeskunde van het VUmc opdrachtgever is van het onderzoek met de titel: "*Less is more: glucose lowering drugs in older people with type 2 diabetes*" (optimalisatie medicatie en diabetes type 2, OMED2)(hierna: het "**Onderzoek**") onder leiding en verantwoordelijkheid van: Prof. dr. P.J.M. Elders (de "**Onderzoeksleider**");
- Het onderzoek wordt uitgevoerd in samenwerking met de afdeling Public Health and Primary Care van Universitair Medisch Centrum Leiden en stichting PHARMO-STIZON.
- De METC van VUmc heeft verklaard dat het onderzoek niet valt niet onder de reikwijdte van de Wet Medisch-wetenschappelijk Onderzoek met mensen (WMO) (correspondentienummer 2021.032).
- de Deelnemende Praktijk beschikt over faciliteiten en personeel met de vereiste vaardigheden, ervaring en kennis die nodig zijn om de uitvoering van het Onderzoek door de Deelnemende Praktijk te ondersteunen;
- de Deelnemende Praktijk wenst op verzoek van het VUmc deel te nemen aan het Onderzoek als hierboven gedefinieerd. Deze Studie valt niet onder de *Wet medisch-wetenschappelijk onderzoek met mensen* (WMO).

- het Onderzoek op verzoek van het VUmc mede uitgevoerd zal worden in Deelnemende Praktijk, onder leiding en verantwoordelijkheid van Prof. dr. P.J.M. Elders.

Het bovenstaande in beschouwing genomen komen Partijen als volgt overeen:

1. Uitvoering van het Onderzoek

1.1 De huisartsenpraktijk zal het Onderzoek uitvoeren volgens de instructies, welke zijn aangehecht aan dit contract in **Bijlage 1** (Voorwaarden en taken huisartsenpraktijk), en welke integraal deel uitmaken van deze overeenkomst en de eventueel daarop volgende amendementen, de eventueel door de Subsidieverstrekker financiële bijdrage opgelegde financiële voorwaarden en met inachtneming van alle op het Onderzoek betrekking hebbende wet- en regelgeving, waaronder de Wet op de Geneeskundige Behandelingsovereenkomst (WGBO), de EU Algemene Verordening Gegevensbescherming (AVG), en de richtsnoeren voor Goede Klinische Praktijken, voor zover van toepassing op de aard van het Onderzoek en voorts de voorwaarden van deze overeenkomst.

2. Duur en beëindiging

2.1 Deze overeenkomst treedt in werking op de datum van ondertekening door beide Partijen en blijven van kracht totdat het Onderzoek is voltooid, dan wel totdat het Onderzoek wordt beëindigd in overeenstemming met het volgende lid.

2.2 Elk der Partijen is gerechtigd deze afspraken en het Onderzoek voortijdig te beëindigen of zijn verplichtingen daaronder met onmiddellijke ingang op te schorten als één van de volgende limitatieve omstandigheden zich voordoet:

a. Iedere wezenlijke tekortkoming in de nakoming van deze afspraken door één der Partijen, welke tekortkoming niet binnen dertig (30) dagen nadat de andere Partij hierom heeft verzocht, is verholpen, tenzij de niet-nakoming niet in redelijke verhouding staat tot de voortijdige beëindiging van het Onderzoek;
b. Indien het in het licht van de medische wetenschap of het belang van de proefpersonen niet verantwoord is om het Onderzoek voort te zetten; dan wel de toestemming voor het Onderzoek door de Medisch Ethische Commissie van VUmc is/wordt ingetrokken,
c. Als een Partij failliet is verklaard of als er een aanvraag tot faillissement tegen deze Partij is aangevraagd;
d. Als de Deelnemende Praktijk Onderzoeker niet langer als de onderzoeker kan optreden (ongeacht de reden) en er geen wederzijds acceptabele vervanging kan worden gevonden binnen redelijke termijn.

2.3 De artikelen 4, 5, 6 ,7 en 8 van dit contract blijven na het aflopen of de beëindiging van de overeenkomst van kracht totdat de rechten en verplichtingen daaruit voortvloeiend zijn vervuld, of zoveel langer als de aard van de bepaling met zich meebrengt.

2.4 Na een besluit tot voortijdige beëindiging van de overeenkomst zullen de Partijen onverwijld het Onderzoek stopzetten op een wijze die medisch verantwoord is voor alle proefpersonen.

3. Financiële Compensatie

3.1 De Deelnemende Praktijk ontvangt een vergoeding voor deelname aan het onderzoek van 200€ (deze vergoeding is inclusief BTW, indien van toepassing).

3.2 Tevens ontvangt de Deelnemende Praktijk een vergoeding voor deelname aan zowel de nascholingen als de intervisiebijeenkomsten. Deze vergoeding bedraagt voor een Huisarts van de Deelnemende Praktijk of een POH respectievelijk €50,- (vijftig euro) en € 25,- (vijfentwintig euro) per uur. Ook deze vergoeding is (indien van toepassing) inclusief BTW.

4. Intellectuele Eigendomsrechten

4.1 Deze overeenkomst heeft geen gevolgen voor (intellectuele eigendoms)rechten op informatie, gegevens, en andere zaken en knowhow die voor de aanvang van het Onderzoek aan één der Partijen toebehoorden of die na de aanvang van het Onderzoek zijn ontwikkeld maar die geen betrekking hebben op het onderwerp van het Onderzoek.

4.2 Het VUmc verkrijgt alle rechten, waaronder intellectuele eigendomsrechten en knowhow (met uitzondering van knowhow inzake klinische procedures en verbeteringen betreffende klinische procedures van de Deelnemende Praktijk en de Huisarts), die direct voortvloeien uit het Onderzoek (hierna: de "**Resultaten**") en stelt de Resultaten ter beschikking aan al haar deelnemende praktijken in het kader van het algemeen maatschappelijk belang met als doel dat deze resultaten vrij toegankelijk zullen zijn voor nieuw wetenschappelijk onderzoek of onderzoeken.

4.3 De Deelnemende Praktijk blijft te allen tijde gerechtigd om haar eigen data en eigen ontwikkelde knowhow te gebruiken voor intern niet-commercieel onderzoek, patiëntenzorg en/of onderwijs al dan niet in samenwerking met niet-commerciële partijen.

5. Vertrouwelijkheid

5.1 Partijen kunnen in het kader van het Onderzoek vertrouwelijke informatie van elkaar ontvangen. De ontvangende Partij zal alle vertrouwelijke informatie van de verstrekkende Partij strikt vertrouwelijk behandelen. De verstrekkende Partij zal de vertrouwelijke informatie als zodanig kenmerken. Zo zal de ontvangende Partij niet zonder schriftelijke toestemming van de verstrekkende Partij vooraf de ontvangen vertrouwelijke informatie aan een derde openbaren of ter beschikking stellen. De ontvangende Partij zal de ontvangen vertrouwelijke informatie uitsluitend gebruiken voor de uitvoering van het Onderzoek.

5.2 De verplichtingen uit artikel 5.1 blijven van kracht gedurende de looptijd van deze overeenkomst en een periode van vijf (5) jaar daarna. De geheimhoudingsverplichting is niet van toepassing voor gegevens welke:

a) reeds aantoonbaar in het bezit zijn van de ontvangende Partij op het moment van ontvangst door ontvangende Partij, of
b) op het moment van ontvangst reeds algemeen bekend zijn, of later bekend worden, zonder dat dit het gevolg is van enig verwijtbaar handelen of nalaten van ontvangende Partij, of
c) voor zover bekend bij de ontvangende Partij op rechtmatige wijze uit andere bron in bezit zijn gekomen van ontvangende Partij.
d) zelfstandig - en zonder gebruik van de vertrouwelijke informatie van de verstrekkende Partij - door ontvangende Partij is ontwikkeld.

6. Publicatie

6.1 VUmc zal de Resultaten van het Onderzoek in samenwerking met UMC Leiden en Pharmo-STIZON in het kader van het algemeen maatschappelijk belang, publiceren in onder meer wetenschappelijke tijdschriften en vakbladen met inachtneming met hetgeen in dit artikel is bepaald.

6.2 De Onderzoeksleider zal elke publicatie van de Resultaten coördineren en waar nodig afspraken hierover maken met de deelnemende praktijken. Eventueel co-auteurschap zal worden vastgesteld op basis van de voor de beroepsgroep geldende richtlijnen voor auteurschap, zoals omschreven in Bijlage 1 Voorwaarden en taken huisartsenpraktijk , dan wel nader overeen te komen tussen Partijen.

6.3 In geen geval zal publicatie van Resultaten door de Deelnemende Praktijk in een ander manuscript aan de hoofdpublicatie voorafgaan, tenzij de hoofdpublicatie twaalf (12) maanden na beëindiging van het Onderzoek is uitgebleven. Na het verstrijken van voorgenoemde periode zal het de Onderzoeker Deelnemende Praktijk vrij zijn om te publiceren conform het bepaalde in artikel 6.4.

6.4 Met inachtneming van het hierboven vermelde, zijn de Partijen gerechtigd de door henzelf gegenereerde Resultaten te publiceren in wetenschappelijke tijdschriften en/of op congressen. Voor een dergelijke publicatie door de Deelnemende Praktijk, zal VUmc in de gelegenheid gesteld worden van de inhoud van de voorgestelde publicatie kennis te nemen. Daartoe zal dertig (30) dagen voor aanbieden van de publicatie het voorstel ter beschikking worden gesteld aan het VUmc. Binnen deze periode zal worden aangeven of er bezwaar is tegen publicatie om een van de hieronder beschreven redenen:

a. vertrouwelijke informatie van VUmc dreigt te worden geopenbaard. Op verzoek van het VUmc zal alle vertrouwelijke informatie uit de voorgenomen publicatie worden verwijderd;

b. de voorgenomen publicatie bevat niet-beschermde octrooieerbare informatie. In dat geval wordt ingestemd met het eventueel uitstellen van het indienen van de publicatie, teneinde het VUmc de mogelijkheid te geven de belangen en rechten van het Project te beschermen. Dit uitstel bedraagt maximaal negentig (90) dagen vanaf het moment dat het manuscript aan VUmc ter beschikking is gesteld.

7. Bescherming persoonsgegevens

7.1 In het kader van het Onderzoek kan de Deelnemende Praktijk persoonsgegevens, al dan gepseudonimiseerd, van betrokken proefpersonen (hierna: de "**Persoonsgegevens"**) verstrekken aan VUmc.

7.2 In lijn met het standpunt van de CCMO, gaan Partijen ervan uit, dat zij met het oog op het Onderzoek zullen moeten worden beschouwd als gezamenlijke verwerkingsverantwoordelijken in de zin van artikel 26 lid 1 AVG, totdat of tenzij de wet of een gewijzigd standpunt van de CCMO en/of een interpretatie van de Autoriteit Persoonsgegevens en/of een rechterlijke uitspraak Partijen dwingt, of erop wijst, de rollen van Partijen in het Onderzoek anders te kwalificeren. In dat geval zullen Partijen met elkaar overleggen en zij zullen de kwalificatie van hun rollen aanpassen en deze regeling wijzigen als passend zal worden geacht in overeenstemming met het bepaalde in de AVG en de door betrokkenen verstrekte toestemming.

7.3 Beide Partijen zullen de Persoonsgegevens op behoorlijke en zorgvuldige wijze en in overeenstemming met de op hen als verantwoordelijken op grond van de AVG rustende verplichtingen verwerken. Verantwoordelijken verstrekken elkaar over en weer alle benodigde informatie teneinde een goede naleving van de relevante wet- en regelgeving mogelijk te maken. Partijen hebben de over te dragen gegevens en de uit de AVG voortvloeiende taken en verantwoordelijkheden gespecificeerd en verdeeld zoals is opgenomen in **Bijlage 2**.

7.4 Partijen, als gezamenlijk verwerkingsverantwoordelijken, zullen met elkaar samenwerken, uit welke samenwerking duidelijk zal blijken welke rol de gezamenlijke verwerkingsverantwoordelijken respectievelijk vervullen, en wat hun respectieve verhouding met de proefpersonen in het Onderzoek (betrokkenen in de zin van de AVG) is, met name met betrekking tot de uitoefening van de rechten van die proefpersonen en hun respectieve verplichtingen bedoeld in de artikelen 13 en 14 AVG. Zowel VUmc als de Deelnemende Praktijk zullen een register bijhouden van de verwerkingsactiviteiten, die onder hun verantwoordelijkheid plaats zullen vinden. Elk van Partijen zal ook overigens de ingevolge de AVG op een verwerkingsverantwoordelijke rustende verplichtingen nakomen jegens proefpersonen in verband met de verwerking van de in het kader van het Onderzoek verzamelde Persoonsgegevens, die hij beheert en verwerkt. Partijen treffen elk passende

technische en organisatorische maatregelen om te waarborgen en te kunnen aantonen dat die verwerking in overeenstemming met de AVG wordt uitgevoerd. Die maatregelen worden geëvalueerd en indien nodig geactualiseerd. Partijen zullen de in het kader van het Onderzoek verzamelde gegevens slechts gebruiken en openbaar maken in overeenstemming met het bepaalde in de AVG en de door betrokkenen verstrekte toestemming.

7.5 Ingeval van een inbreuk op de beveiliging van de Persoonsgegevens, zoals bedoeld in de artikelen 33 en 34 AVG, zullen Partijen onverwijld met elkaar in overleg treden en afstemmen over welke Partij(en) op welke wijze zal voldoen aan de meldingsplichten vermeld in de meldplicht datalekken regelgeving. Voorts erkennen Partijen passende technische en organisatorische maatregelen ten uitvoer te leggen om Persoonsgegevens te beveiligen tegen verlies of tegen enige vorm van onrechtmatige verwerking. Deze maatregelen garanderen, rekening houdend met de stand van de techniek en de kosten van de tenuitvoerlegging, een passend beveiligingsniveau gelet op de risico's die de verwerking en de aard van de te beschermen gegevens met zich meebrengen. De maatregelen zijn er mede op gericht onnodige verzameling en verdere verwerking van persoonsgegevens te voorkomen. In dit verband geldt dat elke Partij verantwoordelijk is voor de beveiliging van de Persoonsgegevens die zich op zijn locatie en op zijn IT-middelen en Infrastructuur bevinden.

7.6 Indien een betrokkene bij één van beide Partijen een verzoek indient tot het uitoefenen van zijn rechten onder de AVG, stelt die partij de wederpartij daarvan onverwijld in kennis. In een dergelijk geval zullen Partijen volledig met elkaar samenwerken om aan het verzoek te kunnen voldoen.

8. Verzekering en vrijwaring

8.1 Elk der Partijen verplicht zich afdoende te verzekeren ter afdekking van schade voortvloeiend uit zijn aansprakelijkheid op grond van de in de overeenkomst gemaakte afspraken en ter zake enige niet-contractuele verbintenis voortvloeiende uit of verband houdende met deze overeenkomst.

8.2 Alle aansprakelijkheid voor indirect- of gevolgschade, hieronder mede begrepen gederfde winst, gederfde omzet en andere inkomsten en gemiste contracten, is te allen tijde uitgesloten, tenzij sprake is van opzet of grove schuld van de aansprakelijke Partij.

9. Toepasselijk recht en jurisdictie

9.1 Deze overeenkomst en alle overeenkomsten en verbintenissen die daaruit voortvloeien of daarmee verband houden, worden beheerst door Nederlands recht.

9.2 De rechtbank te Amsterdam is bij uitsluiting bevoegd om alle geschillen, die het gevolg zijn van of verband houden met deze overeenkomst en/of de overeenkomsten en verbintenissen zoals bedoeld in voorgenoemde zin en die niet op minnelijke wijze geregeld kunnen worden, in eerste aanleg te beslechten (inclusief een geschil over het bestaan, de rechtsgeldigheid of de beëindiging van deze overeenkomst of ter zake enige niet-contractuele verbintenis voortvloeiende uit of verband houdende met deze overeenkomst).

Aldus overeengekomen en opgemaakt in tweevoud,

Ten getuige waarvan, de gerechtigde vertegenwoordigers van Partijen deze afspraken hebben ondertekend, voor:

VUmc

Naam:

Functie:

Datum:

Handtekening:

Namens de deelnemende Praktijk (praktijkhoudende huisarts of directeur gezondheidscentrum)

Naam:

Functie:

Datum:

Handtekening:

[Huisarts (als de praktijkhouder niet zelf de coördinerende huisarts is)

Ik verbind mij door ondertekening voor gelezen en akkoord van deze afspraken de taken van de Huisarts uit te voeren en de daarmee samenhangende verplichtingen na te leven

Naam:

Datum:

Handtekening:

Bijlage 1: Voorwaarden en taken huisartsenpraktijk

Voorwaarden

1. Bereidheid tot deelname aan studie
2. Onderzoekscontract getekend met Amsterdam UMC
3. Samenwerkingsovereenkomst en dataverwerkingsovereenkomst met danwel Academisch netwerk Huisartsgeneeskunde UMC Amsterdam, danwel Academisch Huisartsgeneeskunde Leiden (ELAN) danwel met STIZON .
4. Indien dataverwerkingsovereenkomst met STIZON: een papieren of digitale machtiging (beschikbaar in het portaal www.mijn.inszo.nl of te verkrijgen via mail of post) voor de beschikkingstelling van de gecodeerde data aan de onderzoeksgroep

Taken

5. Deelname aan OMED2-nascholingsprogramma. Dit programma bestaat uit één webinar voor de coördinerend huisarts en vier webinars verdeeld over 2 jaar voor de coördinerend POH. Iedere webinar duurt gemiddeld 1 uur.
6. Selectie van voor de studie in aanmerking komende patiënten met gebruik van danwel VIP Calculus tool of INSZO tool. De onderzoeksgroep biedt hier ondersteuning bij.
7. Registratie van die selectie conform de instructies van de onderzoeksgroep.
8. Schriftelijk uitnodigen van patiënten die hiervoor in aanmerking komen tot deelname aan de studie. De onderzoeksgroep biedt hiervoor administratieve ondersteuning aan.
9. Aanpassen van de zorg conform de instructies tijdens de webinar voor zover dit door de praktijkhouder in samenspraak met de POH wenselijk geacht wordt. De POH en de huisarts vullen aan het eind van de nascholing een korte vragenlijst in (duur 5 tot 10 minuten). Deze vragenlijst wordt aan het eind van de studie nogmaals ingevuld.
10. Zowel de Praktijkhouder als de POH kunnen benaderd worden voor een interview door de onderzoekers over het verloop en de effecten van de nascholing en de implementatie ervan in de praktijk.

Bijlage 2: PRIVACY MATRIX

Bijlage Privacy bij de gegevensoverdracht tussen VUmc en Deelnemende Praktijk behorende bij de hoofdovereenkomst (hierna: de "Overeenkomst")

Beschrijving van de gegevensoverdracht:

**Proefpersonen**<br><br>De overgedragen persoonsgegevens betreffen de volgende categorieën datasubjecten:	Er worden alleen gecodeerde persoonsgegevens overgedragen tenzij de betreffende patiënt toestemming voor de overdracht heeft gegeven.
**Doel(en) van de overdracht**<br>De overdracht vindt plaats met de volgende doelstelling:	Optimalisatie medicatie bij mensen met diabetes type 2.
**Categorieën van data**<br>De over te dragen persoonlijke data betreft de volgende categorieën van data:	ICPC codes van de contacten<br><br>ICPC codes van de episoden<br><br>ICPC codes van de problemen<br><br>Type contactregistraties (consulten, telefoontjes, visites)<br><br>Informatie betreffende medicatieafgifte<br><br>Labwaarden en meetgegevens<br><br>Vrije tekst indien vooraf aangegeven met tekststring "OMED2"<br><br>Ziekenhuisopnames, bezoeken eerste hulp en dienstenpost en verpleeghuisopnames

	(eenmalige lijst na afloopt van de studie ingevuld door de praktijk)<br><br>Behoudens deze vragenlijst worden de gegevens verstrekt door (doorhalen wat niet van toepassing is): Academisch Netwerk Huisartsen Amsterdam (ANHA)/ Stichting PHARMO-STIZON
**Gevoelige data** (indien van toepassing)<br>De over te dragen persoonlijke data betreffen de volgende categorieën van gevoelige data:	Geen
**Methode van overdracht**	De gecodeerde gegevens betreffen digitale extracties uit het elektronische patiëntendossier. De overdracht wordt uitgevoerd door ANHA/STIZON.
**Methode van dataopslag en beveiligingsmaatregelen (bijv. Methode van encryptie)**	De data worden opgeslagen op de M-schijf van VUmc en worden met een wachtwoord beveiligd.
**Geautoriseerde sub-verwerkers**	STIZON/PHARMO<br><br>Leiden Medisch Centrum

Privacy afspraken en toedeling verantwoordelijkheden

**VUmc en Deelnemende Praktijk zijn gezamenlijke verwerkingsverantwoordelijken ten aanzien van de Persoonsgegevens die zullen worden verwerkt en stellen hierbij conform artikel 26 van de AGV hun respectieve plichten ten aanzien van nakoming van de AVG vast.**	
Privacy verantwoordelijkheden	*Verantwoordelijke Partij alsmede nadere specificering van de bijbehorende Persoonsgegevens en de verwerkingsactiviteiten alsmede de wijze van nakoming.*

**1. Informatieplicht over de verwerking van de Persoonsgegevens jegens betrokkenen (artikel 13, 14 WGBO, artikel 12 WMO).**	De deelnemende praktijken informeren hun patiënten via een vermelding op hun website of via een poster in de wachtkamer dat zij gecodeerde elektronische gegevens verstrekken voor wetenschappelijk onderzoek in het kader van (doorhalen wat van toepassing is): Wetenschappelijk onderzoek van het Academisch Netwerk Huisartsgeneeskunde Amsterdam/ELAN/ wetenschappelijk onderzoek door PHARMO/ wetenschappelijk onderzoek door Amsterdam UMC afdeling huisartsgeneeskunde.<br><br>Daarnaast nodigt de deelnemende Praktijk patiënten die akkoord gaan met medicatiemindering uit voor deelname aan aanvullend onderzoek middels een door het VUmc opgestelde informatiebrief en toestemmingsverklaring.
**2. Waarborgen verkrijging toestemming , informed consent of andere grondslag van verwerking (artikel 6 AVG).**	In deze studie verstrekt de deelnemende verstrekt alleen Persoonsgegevens aan Dataontvanger onder deze Overeenkomst als hiervoor informed consent gegeven is of als hiervoor uitzondering voor het vragen van toestemming is. Dit is door de METC beoordeeld van VUmc beoordeeld.
**3. Waarborgen recht tot inzage, rectificatie, gegevenswisseling, beperking, bezwaar van betrokkenen (de artikelen 15 tot en met 18 en artikel 21 AVG).**	Als betrokkene zijn rechten zoals genoemd in de hiernaast opgesomde artikelen inroept, consulteert de Deelnemende Praktijk met het VUmc over de wijze waarop hieraan gevolg zal worden gegeven in overeenstemming met de AVG alsmede de Uitvoeringswet AVG.
**4. Waarborgen recht op Overdraagbaarheid (dataportabiliteit) van betrokkenen (artikel 20 AVG).**	Niet van toepassing
**5. Waarborgen beveiliging van de Persoonsgegevens conform de AVG (artikel 32 AVG) en conform overige afspraken in deze Overeenkomst.**	Deelnemende Praktijk zal de beveiliging van persoonlijke gegevens waarborgen in overeenstemming met artikel 32 AVG zoals opgenomen in deze Overeenkomst. VUmc verwerkt de gegevens conform de NEN 7510.
**6. Waarborgen voldoen aan datalek meldplichten (artikelen 33 en 34 AVG).**	VUmc en Deelnemende Praktijk zullen voldoen aan de verplichtingen inzake inbreuk op de gegevens in overeenstemming met de artikelen 33 en 34 van de AVG. In geval van een (mogelijke) inbreuk zullen Partijen volledig met

	elkaar samenwerken om de inbreuk in verband met persoonsgegevens te verhelpen, tijdig aan de (wettelijke) meldingsverplichtingen te voldoen en de schade te vergoeden.
**7. Waarborgen dat medewerkers die toegang hebben tot de Persoonsgegevens, afdwingbaar zijn geïnstrueerd conform art 29 AVG, waaronder het contractueel opleggen van een geheimhoudingsplicht aan de medewerkers en toezien op naleving van de instructies door deze medewerkers.**	Alle personen die in contact komen met persoonsgegevens hebben een vertrouwelijkheidsverklaring getekend. Hier wordt een logboek van bijgehouden.
**8. Waarborgen ingeschakelde verwerkers die toegang hebben tot de Persoonsgegevens, afdwingbaar zijn geïnstrueerd, waaronder het sluiten van verwerkersovereenkomsten met de verwerkers en toezien op naleving van de instructies door deze verwerkers e.e.a. conform artikel 28 AVG.**	Niet van toepassing.
**9. Waarborgen dat doorgifte van Persoonsgegevens geschiedt conform de AVG.**	Niet van toepassing.
**10. Waarborgen bewaren en vernietigen van Persoonsgegevens.**	VUmc kan de persoonsgegevens tot tien jaar na afloop van de studie opslaan. Daarna zullen deze verwijderd worden. Dit in verband met dat er gelegenheid moet zijn om de wetenschappelijk deugdelijkheid te toetsen.
**11. Waarborg dat uitvoering Privacy Impact Assessment (PIA) voorafgaand aan het verzamelen, waaronder verkrijgen en de verdere verwerking van de Persoonsgegevens (artikel 35 AVG).**	De studie is als niet-WMO afgegeven en door METC van VUmc getoetst op AVG-aspecten.
**12. Overige afgesproken privacy verantwoordelijkheden.**	Indien de afspraken die zijn opgenomen in deze matrix/bijlage onvolledig of onjuist blijken te zijn, zullen de partijen deze matrix zodanig aanpassen dat ze voldoen aan de AVG.<br><br>De huisarts biedt patiënten een beschrijving van het onderzoek en een informed consent-formulier aan samen met een antwoordenveloppe waarmee het informed consent-formulier naar VUmc kan worden opgestuurd. Op het formulier staat een nummer vermeld. Als er door VUmc een informed consent-formulier wordt ontvangen, dan stuurt VUmc een PDF van dit formulier naar de huisarts terug. VUmc verifieert het sequentienummer bij de huisarts. De huisarts

	draagt er zorg voor dat het nummer met de tekstring OMED2 ervoor in het elektronische dossier van de betreffende patiënt wordt vermeld waardoor de persoonsgegevens aan het gecodeerde elektronische dossier kunnen worden gekoppeld door VUmc.
