# Supplementary material for: A deprescribing programme aimed to optimise blood glucose-lowering medication in older people with type 2 diabetes mellitus, the OMED2-study: the study protocol for a randomised controlled trial
Source: Trials. 2024 Jul 25;25:505. doi: 10.1186/s13063-024-08249-9 (PMC11271055; doi:10.1186/s13063-024-08249-9)
Supplement: Supplementary file 4 — Supplementary Material 4. [file 13063_2024_8249_MOESM4_ESM.pdf]

STIZON

3. Machtiging beschikbaarstelling huisartsdata aan derden voor OMED2 studie

Ondergetekende heeft een verwerkers- en dienstverleningsovereenkomst met Stichting Informatievoorziening voor Zorg en Onderzoek (STIZON), waarin toestemming wordt verleend om voor onderzoek- en rapportagedoeleinden benodigde gegevens periodiek uit het Zorginformatiesysteem te downloaden en deze gegevens via datatransfer te plaatsen in de beveiligde STIZON omgeving.

Ondergetekende heeft een verwerkingsovereenkomst met (Amsterdam Universitair Medisch Centrum (Amsterdam UMC)

Ondergetekende machtigt hierbij STIZON tot het uitvoeren van de volgende werkzaamheden:

- **vier** keer extraheren van uw huisartseninformatiesysteem (HIS) voor het **'Ouderen met medicatie diabetes type2' (OMED2) project.**
- verstrekken identificerende en medische gegevens aan INSZO (Instituut voor Zorgoptimalisatie), Van Deventerlaan 30-40, 3528 AE Utrecht, voor het maken van terugrapportages voor de eigen praktijk binnen de studie **'Optimalisatie medicatie diabetes type2' (OMED2)** van Amsterdam UMC (Amsterdam Universitair Medisch Centrum) en LUMC (Leids Universitair Medisch Centrum).
- ter beschikking stellen van **geanonimiseerde patiëntgegevens** aan **Amsterdam UMC en LUMC (Leids Universitair Medisch Centrum)**, ten bate van de OMED2 studie.
- Na het tekenen van een OMED2 **informed consent** door de patiënt stelt STIZION **herleidbare patiëntgegevens aan Amsterdam UMC (Amsterdam Universitair Medisch Centrum) en LUMC (Leids Universitair Medisch Centrum)**.

De geldigheidsduur van deze machtiging is gekoppeld aan de looptijd van bij 10.1 genoemde Verwerkersovereenkomst en stopt automatisch bij de beëindiging hiervan. Ondergetekende kan de machtiging ook tussentijds beëindigen, door intrekking van de machtiging schriftelijk aan Stichting Informatievoorziening voor Zorg en Onderzoek te melden.

Ondergetekende verklaart en garandeert uitdrukkelijk dat hij/zij volledig bevoegd is tot het verstrekken van deze machtiging.

Naam praktijk:	
AGB code praktijk:	
Software systeem (HIS):	
Adres:	
Postcode + Plaats:	
Telefoonnummer:	
E-mail adres:	

Naam praktijkhouder(s):	
AGB code(s):	
Plaats:	
Datum:	
Akkoord:	*(handtekening(en))*
